# Biallelic Variants in *MNS1* Are Associated with Laterality Defects and Respiratory Involvement

**DOI:** 10.3390/cells13121017

**Published:** 2024-06-11

**Authors:** Rim Hjeij, Joseph Leslie, Hoda Rizk, Bernd Dworniczak, Heike Olbrich, Johanna Raidt, Sebastian Felix Nepomuk Bode, Alice Gardham, Karen Stals, Mohammad Al-Haggar, Engy Osman, Andrew Crosby, Tarek Eldesoky, Emma Baple, Heymut Omran

**Affiliations:** 1 Department of General Pediatrics, University Hospital Muenster, 48149 Muenster, Germany; bernd.dworniczak@t-online.de (B.D.); heike.olbrich@ukmuenster.de (H.O.); johanna.raidt@ukmuenster.de (J.R.); heymut.omran@ukmuenster.de (H.O.); 2 Institute of Biomedical and Clinical Science, RILD Wellcome Wolfson Centre, University of Exeter Medical School, Royal Devon University Healthcare NHS Foundation Trust, Exeter EX2 5DW, UK; j.leslie@exeter.ac.uk (J.L.); a.h.crosby@exeter.ac.uk (A.C.); e.baple@exeter.ac.uk (E.B.); 3 Department of Pediatrics, Faculty of Medicine, University of Mansoura, Mansoura 35516, Egypt; hodarizk@mans.edu.eg (H.R.); engyosman73@mans.edu.eg (E.O.); tarekdag@mans.edu.eg (T.E.); 4 Department of Pediatrics, University Hospital Ulm, 89075 Ulm, Germany; sebastian.bode@uniklinik-ulm.de; 5 North West Thames Regional Genetic Service, North West London Hospitals, London HA1 2UJ, UK; alice.gardham@nhs.net; 6 Exeter Genomics Laboratory (NHS South West Genomic Laboratory Hub), Royal Devon University Healthcare NHS Foundation Trust, Exeter EX2 5DW, UK; karen.stals@nhs.net; 7 Genetics Unit, Pediatrics Department, Faculty of Medicine, Mansoura University, Mansoura 35516, Egypt; malhaggar@mans.edu.eg; 8 Peninsula Clinical Genetics Service, Royal Devon & Exeter Hospital (Heavitree), Exeter EX1 2ED, UK

**Keywords:** ciliopathy, situs inversus, primary ciliary dyskinesia, MNS1

## Abstract

Defects in motile cilia, termed motile ciliopathies, result in clinical manifestations affecting the respiratory and reproductive system, as well as laterality defects and hydrocephalus. We previously defined biallelic *MNS1* variants causing *situs inversus* and male infertility, mirroring the findings in *Mns1*^−/−^ mice. Here, we present clinical and genomic findings in five newly identified individuals from four unrelated families affected by *MNS1*-related disorder. Ciliopathy panel testing and whole exome sequencing identified one previously reported and two novel *MNS1* variants extending the genotypic spectrum of disease. A broad spectrum of laterality defects including *situs inversus totalis* and heterotaxia was confirmed. Interestingly, a single affected six-year-old girl homozygous for an *MNS1* nonsense variant presented with a history of neonatal respiratory distress syndrome, recurrent respiratory tract infections, chronic rhinitis, and wet cough. Accordingly, immunofluorescence analysis showed the absence of MNS1 from the respiratory epithelial cells of this individual. Two other individuals with hypomorphic variants showed laterality defects and mild respiratory phenotype. This study represents the first observation of heterotaxia and respiratory disease in individuals with biallelic *MNS1* variants, an important extension of the phenotype associated with MNS1-related motile ciliopathy disorder.

## 1. Introduction

Ciliopathies are a group of heterogeneous hereditary disorders resulting from abnormal ciliary structure and/or function. Cilia are filamentous organelles, expressed on almost every cell type of the human body, that play pivotal roles in various physiological processes across different organs and tissues [1]. Traditionally, cilia have been categorized based on their structure and function: primary singular immotile cilia are implicated in sensory perception and signaling pathways whereas motile cilia fulfill important roles in mucociliary clearance and the establishment of left–right body asymmetry as well as facilitating fertility in both males and females [2,3]. Motile and immotile cilia can have either a 9 + 0 or a 9 + 2 ultrastructure [4]. The 9 + 0 structure consists of 9 outer doublet microtubules without a central pair, while the 9 + 2 structure consists of 9 outer doublet microtubules surrounding a central pair (CP) of microtubules. Outer dynein arms (ODAs) and inner dynein arms (IDAs) are motor proteins attached to the doublet microtubules generating the force required for ciliary beating, with ODAs primarily responsible for the power stroke and IDAs regulating the beating pattern and frequency [5]. Consequently, the implications of ciliary defects are wide-ranging, leading to a spectrum of manifestations depending on the type of cilia affected. For instance, dysfunctional respiratory cilia can lead to recurrent respiratory infections, while anomalies in nodal cilia may cause laterality defects. Similarly, defects in ependymal cilia are associated with hydrocephalus, and issues with flagella can result in male infertility. Primary ciliary dyskinesia (PCD) is a disorder typified by the presence of otosinopulmonary disease, often accompanied by male infertility and laterality defects, which affects approximately 1 in 15,000 to 1 in 20,000 individuals worldwide [6,7]. When laterality defects occur together with the above clinical symptoms, we refer to this condition as Kartagener’s syndrome [8]. One of the hallmark features of Kartagener syndrome is situs inversus, a condition in which the major visceral organs are mirrored from their normal positions [9,10]. Diagnosing PCD is multifaceted, involving a suite of diagnostic tools. In addition to clinical symptoms, PCD diagnosis may require nasal nitric oxide measurements (nNO) (documented to be low in PCD-affected individuals), high-speed video microscopy measuring the ciliary beat frequency and waveform, transmission electron microscopy revealing the ciliary ultrastructural defects, and genetic analysis revealing a compatible molecular diagnosis [11]. In recent years, genetic analysis has become a cornerstone of PCD diagnosis, enabling the identification of causative variants. Lately, immunofluorescence analysis revealing the molecular ciliary defects has been extensively used to confirm the pathogenicity of the identified variants as well as to decipher the relationship of specific proteins to each other within defined ciliary complexes [12,13,14], and air–liquid ciliary cultures have been developed as an aid to diagnostic testing of PCD [13].

To date, more than 60 genes have been reported with pathogenic variants associated with motile ciliopathy disorders [1,12]. While most of these genes are primarily associated with respiratory symptoms, recently, a small subset of these genes have been described in association with laterality defects and male infertility but with mild or no respiratory symptoms. Notable among these are pathogenic variants in genes such as *CCDC11/CFAP53*, *ENKUR*, *WDR16/CFAP52*, *DAW1*, and *MNS1* [15,16,17,18,19,20,21]. These discoveries highlight the diverse impact of ciliary dysfunction beyond respiratory disease, emphasizing the importance of cilia in body asymmetry and reproductive health.

*MNS1*, a meiosis-specific nuclear structural 1 protein, was initially identified in the proteomes of human bronchial epithelium [22] and during spermatogenesis [23], and is reported to have an essential role in motile ciliary function and sperm flagella assembly in mice [24]. We subsequently identified homozygous nonsense variants in *MNS1* as a cause of *situs inverus* and male infertility in three families [21]. More recently, we reported a homozygous founder frameshift variant in *MNS1* in four inter-related families of Amish descent, in association with randomization of left–right body asymmetry and male infertility [25], and a homozygous frameshift variant was reported in a Han Chinese male with oligoasthenoterato-zoospermia [26].

The current study identifies a further four affected individuals and a fetus with *MNS1*-related motile ciliopathy from four families from Afghanistan, Egypt, and Kosovo. All four affected individuals presented with laterality defects, including one male individual with heterotaxia and complex congenital heart defects. In addition, one of the affected children displayed a pronounced respiratory phenotype not previously seen in *MNS1*-related disease. This observation is particularly significant as it suggests a more extensive role of this gene in ciliary functions beyond its previously identified implications in laterality and fertility.

## 2. Materials and Methods

### 2.1. Human Samples

For the purpose of this study, biological specimens including nasal brush biopsies and blood samples were collected from three distinct groups: affected individuals displaying clinical symptoms, family members, and healthy control individuals. Ciliated respiratory cells from nasal brush biopsies were used for immunofluorescence (IF) microscopy analyses. All the details of the affected individuals included in this study are summarized in Table 1.

### 2.2. Genomic Studies

Genomic DNA was extracted from blood samples by standard methodology from blood. Genomic investigations in OP-4846II1, OP-4761II1, and OP-4242II1 comprised ciliopathy gene panel sequencing. The panel includes all published PCD and motile ciliopathy-related genes (see the below table for details). Variants present in the dbSNP database, the 1000 Genomes Project polymorphism, and the Genome Aggregation Database (gnomAD V4.0) with a minor-allele frequency > 0.01% were excluded. Nonsynonymous variants, variants impacting the consensus splice sites, and insertions/deletions (indels) consistent with an X-linked or autosomal recessive inheritance pattern were prioritized for analysis.

For family EX-1 (individual EX-1II1), trio whole exome sequencing was performed on Illumina platforms as previously described (PMID: 33845882). Briefly, WES (NextSeq 500; Illumina v2.5, Twist Human Core Exome targeting) involved read alignment (BWA-MEM v0.7.17), mate-pairs fixed and duplicates removed (Picard v2.15), InDel realignment and base quality recalibration (GATK v3.8.0), single nucleotide variant and InDel detection (GATK HaplotypeCaller; HaplotypeCaller–GATK (broadinstitute.org)), and variant annotation (Alamut batch v1.11). Read depth was determined for the whole exome through our in-house pipeline. Copy number variants (CNVs) were detected using SavvyCNV (https://github.com/rdemolgen/SavvySuite). Variants were prioritized by call quality, inheritance pattern, allele frequency, and predicted functional consequence and correlated with the clinical phenotype.

For all families, unique primers were utilized for amplification and bidirectional Sanger sequencing of the *MNS1* variants to confirm the segregation of the variant with the disease phenotype.

### 2.3. Genes Included in Targeted PCD Gene Panel Sequencing

*ARMC4* (NM_001290020, NM_018076), *CCDC39* (NM_181426), *CCDC40* (NM_017950, NM_001330508, NM_001243342), *CCDC65* (NM_033124), *CCDC103* (NM_213607), *CCDC114* (NM_144577), *CCDC151* (NM_145045), *CCNO* (NM_021147), *CFAP43* (NM_025145), *CFAP44* (NM_001164496), *CFAP45* (NM_012337), *CFAP52* (NM_145054), *CFAP53* (NM_145020), *CFAP69* (NM_001160138, NM_001039706), *CFAP70* (NM_001367801), *CFAP74* (NM_001304360), *CFAP221* (NM_001271049), *CFAP298/C21orf59* (NM_001350335, NM_021254), *CFAP300/C11orf70* (NM_032930), *DNAAF1* (NM_178452, NM_001318756), *DNAAF2* (NM_018139), *DNAAF3* (NM_001256714), *DNAAF4* (NM_130810, NM_001033560), *DNAAF5* (NM_017802), *DNAAF6/PIH1D3* (NM_001169154, NM_173494), *DNAAF11/LRRC6* (NM_012472, NM_001321965), *CFTR* (NM_000492), *DNAH1* (NM_015512), *DNAH2* (NM_020877; NM_001303270), *DNAH5* (NM_001369), *DNAH8* (NM_001206927), *DNAH9* (NM_001372), *DNAH10* (NM_207437), *DNAH11* (NM_001277115), *DNAI1* (NM_001281428), *DNAI2* (NM_001353167), *DNAJB13* (NM_153614), *DNAL1* (NM_031427, NM_001201366), *DRC1* (NM_145038), *DYNC2H1* (NM_001080463), *ENKUR* (NM_145010, NM_001270383), *FOXJ1* (NM_001454), *FSIP2* (NM_173651), *GAS2L2* (NM_139285), *GAS8* (NM_001481), *HYDIN* (NM_001270974, NM_001198542), *INVS* (NM_014425), *LRRC56* (NM_198075), *MCIDAS* (NM_001190787), *MNS1* (NM_018365), *NEK10* (NM_152534, NM_001304384), *OFD1* (NM_003611, NM_001330210), *RPGR* (NM_000328, NM_001034853), *RSPH1* (NM_080860), *RSPH3* (NM_031924), *RSPH4A* (NM_001010892), *RSPH9* (NM_001193341), *SPAG1* (NM_003114, NM_172218), *SPEF2* (NM_024867), *STK36* (NM_015690), *TP73* (NM_005427, NM_001126240, NM_001204192), *TTC12* (NM_001318533, NM_001352038, NR_147891), *TTC25* (NM_031421), *TXNDC3/NME8* (NM_016616), and *ZMYND10* (NM_015896).

### 2.4. Immunofluorescence Analysis of Human Respiratory Cells

The procedure for immunofluorescence analysis on human respiratory cells entailed methodologies previously established in the field [27]. We utilized a polyclonal rabbit anti-MNS1 antibody (HPA039975), procured from Atlas Antibodies, to detect the MNS1 protein within the cells. The specificity of this antibody was ascertained through Western Blot analysis, ensuring its reliability for our study. For the detection of primary antibodies, we employed secondary antibodies conjugated with Alexa Fluor dyes: anti-mouse Alexa Fluor 488 and anti-rabbit Alexa Fluor 546, sourced from Molecular Probes by Invitrogen (Life Technologies, Darmstadt, Germany). To visualize cell nuclei, we stained the DNA with Hoechst 33342, a compound obtained from Sigma (Merck, Darmstadt, Germany). Imaging of the stained cells was carried out using a Zeiss Apotome Axiovert 200 microscope (Oberkochen, Germany), and the acquired images were subsequently processed and analyzed using AxioVision 4.8, ZEN software, and Adobe Creative Suite 4, facilitating detailed examination and documentation of cellular features pertinent to our study.

## 3. Results

**Figure 1:**
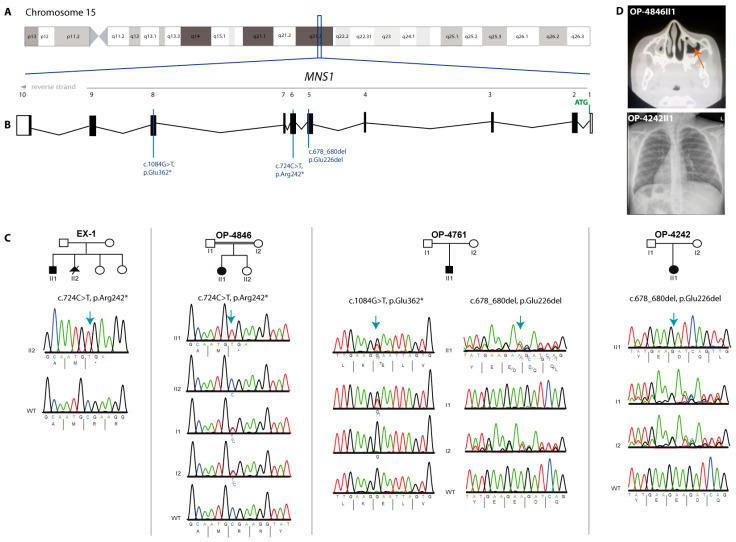
*MNS1* pathogenic variants in individuals with laterality defects and respiratory symptoms. (**A**,**B**). Schematic of chromosome 15 and transcript structure of *MNS1*. The positions of the identified variants are indicated by blue lines. (**C**) Pedigrees and segregation analyses of four families demonstrate the affected individuals’ biallelic variants in *MNS1* (the blure arrow indicates the position of the variant and * stands for a stop codon). (**D**) CT scan showing mucosal thickening (orange arrow) of both maxillary sinuses consistent with chronic sinusitis in OP-4846II1. Chest X-ray of the upper abdomen of individual OP-4242II1 shows dextrocardia and *situs inversus abdominalis*.

Using either whole exome sequencing (Family EX-1) or targeted PCD gene panel sequencing encompassing all known PCD and motile ciliopathy-related genes at the time of investigation (see Methods for details), we identified three affected males and two affected females associated with three *MNS1* variants (Figure 1A–C).

A homozygous nonsense variant in *MNS1* NM_018365.4:c.724C>T;p.(Arg242*) was identified in OP-4846II1 (Figure 1C), a 6-year-old Egyptian female, and in a 10-year-old male of Afghan origin (individual EX-1II1). This variant segregated in both families in an autosomal recessive manner with parents and an unaffected sister of OP-4846II1 all found to be heterozygous for the variant. OP-4846II1 presented with *situs inversus totalis*. Interestingly, OP-4846II1 had a history of neonatal respiratory distress syndrome (RDS) and was admitted at birth to the neonatal intensive care unit for six days. In infancy, she suffered from chronic rhinitis with nasal congestion and chronic wet cough. Due to recurrent respiratory tract infections, she was admitted once to the hospital for bronchopneumonia (Table 1). Individual EX-1II1 presented with right isomerism, asplenia, and complex congenital heart disease that required surgical intervention. There was no history of respiratory symptoms; however, due to the presence of laterality defects, he was investigated for PCD. Light microscopy of ciliary samples collected from individual EX-1II1 revealed a ciliated epithelium with a normal beat pattern and ciliary beat frequency that was able to clear debris. The parents conceived a further affected male pregnancy (individual EX-1II2). Prenatal anomaly scans identified *situs inversus* and dextrocardia and the pregnancy was subsequently interrupted. Targeted Sanger sequencing of DNA derived from individual EX-1II2 confirmed that they were homozygous for the p.(Arg242*) variant. This variant has previously been reported in Clinvar (VCV000973691.45).

In addition, we identified biallelic *MNS1* variants in two Kosovo individuals, a 1-year-old male (OP-4761II1) and a 14-year-old female (OP-4242II1) (Table 1). OP-4242II1 was homozygous for an inframe deletion of one amino acid in *MNS1* NM_018365.4:c.678_680del; p.(Glu226del) and was affected by *situs inversus totalis*, while OP-4761II1 was also diagnosed with *situs inversus totalis* and carried the same variant in a heterozygous state in addition to a heterozygous nonsense variant NM_018365.4:c.1084G>T; p.(Glu362*), resulting in an early stop codon on the other allele (Figure 1C). Interestingly, OP-4761II1 showed recurrent respiratory symptoms including RDS with transient tachypnoe, rhinitis and rhinosinusitis, recurrent pneumonia, recurrent bronchitis, and protracted cough after infections. The fertility status of OP-4761II1 could not be assessed due to his young age. Parents of OP-4242II1 are heterozygous for the inframe deletion variant and both variants identified in OP-4761II1 are each carried simultaneously by one of the parents, confirming the compound heterozygosity. The p.(Glu226del) variant is present in gnomAD at a low frequency (0.00001033 gnomAD v.4.0.0) as is the p.(Glu362*) variant (0.00001437 gnomAD v.4.0.0); importantly, both are only present in a heterozygous state.

To investigate the impact of the identified variants, we performed immunofluorescence analysis to study the expression and localization of MNS1 in the human respiratory axonemes using antibodies targeting MNS1. The specificity of the antibody was previously reported using SDS–PAGE analysis, showing a single band with the correct size [16].

**Figure 2:**
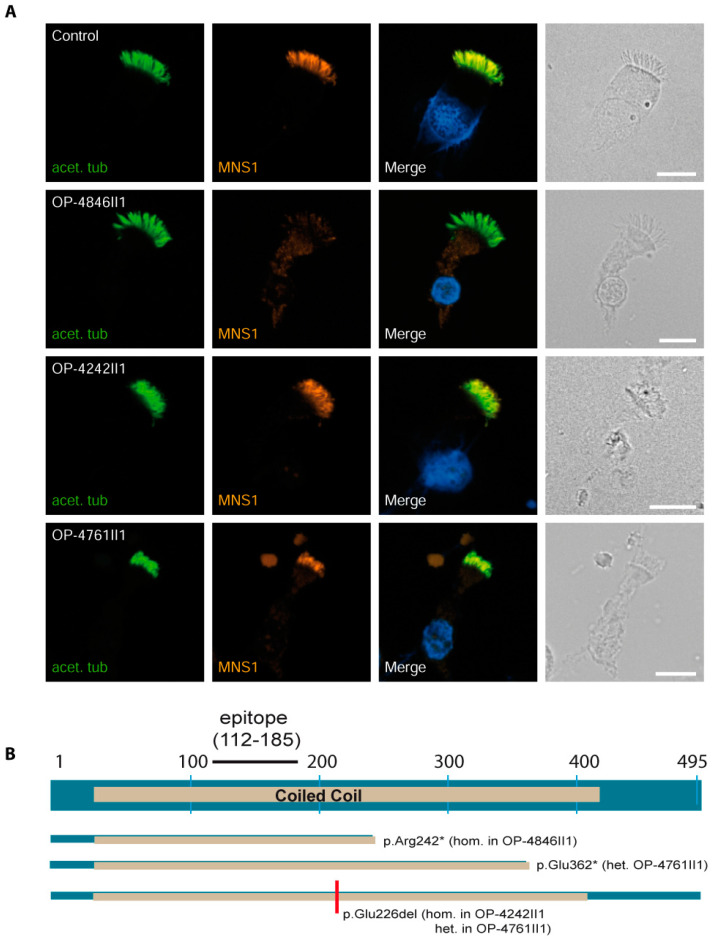
MNS1 is an axonemal ciliary protein that is absent from respiratory cells with pathogenic variants in *MNS1*. (**A**) Respiratory epithelial cells from control and affected individuals OP-4846II1, OP-4242II1, and OP-4761II1 carrying bi-allelic *MNS1* variants are double-labeled with antibodies directed against acetylated α tubulin (green) and MNS1 (red). Nuclei are stained with Hoechst 33342 (blue). In unaffected controls, MNS1 localizes to the entire axonemal length. However, in OP-4846II1, MNS1 is undetectable, consistent with the pathogenic *MNS1* variants identified in this individual. Interestingly, in OP-4242II1 and OP-4761II1, MNS1 still localizes to the axonemes. Scale bars, 10 µm. (**B**) Schematic of the MNS1 protein structure, showing the coiled-coil domains. Only nonsense variants are predicted to lead to a premature termination of translation resulting in truncated proteins, whereas the other variants are in frame and lead to the deletion of one amino acid. The red bar indicates the position of the deletion and * stands for a stop codon.

In control respiratory epithelial cells, MNS1 localized along the length of the ciliary axonemes. MNS1 is a protein with 495 amino acids enclosing a coiled-coil domain from position 28 to 410 (Figure 2B). Individual OP-4846II1 harbors a homozygous NM_018365.4: *MNS1* c.724C>T nonsense variant that results in a premature stop codon at position 242 (p.(Arg242*)) and is expected to undergo nonsense-mediated decay, resulting in loss of function. We used an anti-MNS1 antibody that binds to MNS1 at position 112 to 185 aa to investigate the possibility that the variant transcript escapes nonsense-mediated decay. MNS1 was undetectable in the ciliary axonemes, confirming the expected loss of function due to nonsense-mediated decay of the mRNA transcript. The NM_018365.4:c.1084G>T nonsense variant also resulting in a premature stop codon at position 362 (p.(Glu362*)) was identified only on one allele, inherited from the father. Although this variant would be predicted to undergo nonsense-mediated decay, the other allele carried an inframe deletion p.(Glu226del) outside the binding region of the epitope, and we therefore expected to detect the MNS1 protein in these samples. In respiratory epithelial cells from OP-4761II1 and OP-4242II1, MNS1 could still localize to the ciliary axonemes (Figure 2A), suggesting that the p.(Glu226del) variant is expressed and is able to localize correctly. We therefore hypothesize that the MNS1 protein is still expressed and is stable but likely with reduced function, and therefore we consider the p.(Glu226del) variant identified in these individuals to be hypomorphic.

## 4. Discussion

Motile ciliopathies arising from impaired structure or function of motile cilia are an expanding group of disorders, with PCD remaining the most common presentation. PCD is clinically defined by the presence of otosinopulmonary disease caused by recurrent infections owing to defects in mucociliary clearance, and is associated with laterality defects in about 50% of cases [1,28,29]. Male infertility due to defects in sperm flagella has also been shown to be a common feature [30,31]. Recently, an increasing number of individuals affected by motile ciliopathies have been identified as presenting with mild respiratory phenotypes that are easily overlooked and underdiagnosed, but may still result in a progressive decline in lung function. Therefore, patients presenting with laterality defects such as *situs inversus* or *situs ambiguous* who lack strong otosinopulmonary symptoms should be thoroughly examined for any mild respiratory symptoms. To date, around 30% of PCD cases remain genetically unsolved [25], and mild PCD cases are significantly underdiagnosed. Lately, several genes have been reported to be associated with laterality defects, male infertility, and mild variable respiratory phenotypes such as *CCDC11/CFAP53*, *ENKUR*, *WDR16/CFAP52*, *DAW1*, and *MNS1* [15,16,17,18,19,20].

The MNS1 protein was first discovered during the process of spermatogenesis, as reported by Furukawa et al. in 1994 [23], and in the proteomes of human bronchial epithelium by Ostrowski and colleagues in 2002 [22]. In 2012, Zhou and his team highlighted its critical function in the development of motile cilia and the assembly of sperm flagella in mice. In 2018, we described biallelic pathogenic mutations in *MNS1* associated with laterality defects and infertility in males [21]. Using immunofluorescent staining, it was established that MNS1 is specifically localized to the axonemes of respiratory cilia and sperm flagella in humans. This precise localization suggested a fundamental role for MNS1 in the structural integrity and function of these motile appendages. Further investigation into the ultrastructure of respiratory epithelial cells unveiled a subtle defect in the outer dynein arm (ODA), akin to the abnormalities previously identified in mice lacking Mns1 [21,24]. This defect impacts the efficiency and movement of cilia, crucial for respiratory function and fertility. In a more recent study, we identified a homozygous frameshift founder variant in *MNS1* among four interrelated Amish families, which is associated with irregularities in left–right body asymmetry and male infertility [25]. This was followed by the discovery of a similar homozygous frameshift mutation in a Han Chinese individual suffering from oligoasthenoteratozoospermia by Li et al. in 2021 [26]. Immunostaining techniques demonstrated the typical presence of MNS1 across the entire sperm flagella, yet it was conspicuously absent in the sperm of the proband. This absence was corroborated by Western Blot analysis, which showed no MNS1 protein in the proband’s sperm samples. Moreover, the proband’s sperm exhibited abnormal flagellar morphology and ultrastructural anomalies in the outer doublet microtubules, indicating significant impairments in sperm structure and function, underscoring the profound impact of the MNS1 mutation on fertility outcomes. Despite the attempt to overcome infertility through three rounds of intracytoplasmic sperm injection (ICSI) treatments for the proband’s wife, these interventions did not result in pregnancy.

Most individuals reported with *MNS1*-related motile ciliopathy to date have displayed laterality defects and/or male infertility. We have previously shown that the laterality abnormalities in affected individuals correlate with the expression of Mns1 in the ventral node of mouse embryos [21]. Here, we report an affected individual (OP-4846II1) homozygous for a nonsense variant c.724C>T in *MNS1* resulting in an early stop codon (p.(Arg242*)), where MNS1 is undetectable in the axonemes of respiratory epithelial cells. The *MNS1* c.724C>T, p.(Arg242*) nonsense variant identified in a homozygous state in three individuals in this study has been previously reported in four different male individuals who suffered only from *situs inversus* and infertility. It is likely that this variant represents an Arab founder variant, having now been identified in Palestinian, Jordanian, Egyptian, and Afghan individuals. No females with pathogenic variants in *MNS1* variants have been reported previously aside from a female with PCD that we reported in 2018 who was homozygous for pathogenic variants in *MNS1* as well as in *DNAH5*, an outer dynein arm structural component and one of the most affected genes in PCD [21,32]. Therefore, the respiratory phenotype in this female was contributed to by the deficiency of DNAH5. In this study, we identify for the first time a respiratory phenotype in association with *MNS1*-related motile ciliopathy with two males affected by mild symptoms including RDS, rhinitis, sinusitis, recurrent cough, and a female with a pronounced respiratory phenotype. Anecdotally, we have observed that in motile ciliopathies associated with male infertility, where respiratory involvement is only an infrequent finding and typically mild, females appear to be more affected by respiratory disease than males.

In addition to the roles previously mentioned, through co-immunoprecipitation and yeast two-hybrid assays, we have also shown a significant interaction between MNS1 and CCDC114 [21], which is a component of the outer dynein arm docking complex, abbreviated as ODAD1 [33], indicating a deeper involvement of MNS1 in the structural and functional integrity of cilia and flagella. Our initial description of MNS1 as a cause of laterality defects and male infertility in humans included a particularly revealing finding, whereby ciliary ultrastructural analyses from the female with biallelic pathogenic variants in *MNS1* and *DNAH5* shed light on MNS1’s specific involvement in the docking process of the ODA (ODA-DC) at the distal ends of respiratory axonemes. This suggests that MNS1 is integral to the assembly and stabilization of the ODA, which is essential for the proper movement of cilia.

More recently, research by Ma and Gui in 2019 and 2021 has led to the classification of MNS1 as an inner microtubular protein (MIP) [34,35]. This new classification further underscores the importance of MNS1 in the microtubular architecture within cells, particularly in relation to its role in the assembly and function of motile structures such as cilia and sperm flagella. This dual involvement both in the docking of the dynein arms necessary for ciliary and flagellar movement and as a component of the microtubular structure itself positions MNS1 as a critical player in the maintenance and function of these essential cellular components. Similar to MNS1, CCDC11/CFAP53, also listed as an MIP protein, regulates mammalian cilia-type motility patterns through differential localization and recruitment of axonemal dynein components [36].

## 5. Conclusions

The novel observations of heterotaxia, complex congenital heart disease, and variable respiratory symptoms in patients with biallelic *MNS1* variants highlight the well-established clinical variability of motile ciliopathy disorders. This variability is observed even between individuals harboring the same genetic variant, indicating that clinical presentations are likely influenced by other genetic and environmental factors. The broad phenotypic spectrum associated with ciliary dysfunction emphasizes the critical importance of cilia in maintaining the structural and functional integrity of human physiology. Furthermore, they highlight the necessity for comprehensive genetic screening and diagnosis in patients presenting with symptoms indicative of ciliary dysfunction. As our understanding of the genetic basis of ciliopathies expands, it opens new avenues for the development of targeted therapeutic interventions and enhances our ability to provide accurate diagnoses and management strategies for affected individuals. This study not only contributes to the growing body of knowledge on ciliopathies but also highlights the importance of interdisciplinary research in unraveling the multifaceted roles of cilia in human health and disease.

## Figures and Tables

**Table 1:** Individuals carrying variants in *MNS1*.

ID	Sex	Age	Ethnicity	MNS1 Genotype	Cons	Laterality Defect	Congenital Heart Disease	RDS	Rhino-Sinusitis	Chronic Wet Cough	Bronchiectasis
EX-1II1	M	10y	Afghan	p.(Arg242*)/p.(Arg242*)	Yes	Heterotaxy asplenia	Yes	No	No	No	No
EX-1II2	M	Fetus	Afghan	p.(Arg242*)/p.(Arg242*)	Yes	*Situs inversus*	No	NK	NK	NK	NK
OP-4846II1	F	7y	Egypt	p.(Arg242*)/p.(Arg242*)	Yes	*Situs inversus*	No	Yes	Yes; bilateral maxillary and ethmoidal sinusitis	Yes	No
OP-4761II1	M	2y	Kosovo	p.(Glu362*)/p.(Glu226del)	NK	*Situs inversus*	No	Yes; transient tachypnoe	Yes	No	NK
OP-4242II1	F	15y	Kosovo	p.(Glu226del)/p.(Glu226del)	No	*Situs inversus*	No	No	No	No	NK

F: female; M: male; y: years; cons: consanguinity; RDS: respiratory distress syndrome; NK: not known; *: stop codon

## Data Availability

All data supporting this study are available in the main text and upon request from the corresponding author. Primer sequences for Sanger sequencing were not included in the manuscript but are fully available from the corresponding author upon request.
